# The Effect of PPE-Induced Emphysema and Chronic LPS-Induced Pulmonary Inflammation on Atherosclerosis Development in APOE*3-LEIDEN Mice

**DOI:** 10.1371/journal.pone.0080196

**Published:** 2013-11-26

**Authors:** P. Padmini S. J Khedoe, Man C. Wong, Gerry T. M. Wagenaar, Jaap J. Plomp, Miranda van Eck, Louis M. Havekes, Patrick C. N. Rensen, Pieter S. Hiemstra, Jimmy F. P. Berbée

**Affiliations:** 1 Department of Pulmonology, Leiden University Medical Center, Leiden, The Netherlands; 2 Department of Endocrinology and Metabolic Diseases, Leiden University Medical Center, Leiden, The Netherlands; 3 Department of Pediatrics, Leiden University Medical Center, Leiden, The Netherlands; 4 Department of Neurology, Leiden University Medical Center, Leiden, The Netherlands; 5 Department of Cardiology, Leiden University Medical Center, Leiden, The Netherlands; 6 Einthoven Laboratory for Experimental Vascular Medicine, Leiden University Medical Center, Leiden, The Netherlands; 7 Division of Biopharmaceutics, Leiden Academic Centre for Drug Research, Gorlaeus Laboratories, Leiden, The Netherlands; 8 Netherlands Organization for Applied Scientific Research, Metabolic Health Research, Gaubius Laboratory, Leiden, The Netherlands; Leiden University Medical Center, The Netherlands

## Abstract

**Background:**

Chronic obstructive pulmonary disease (COPD) is characterized by pulmonary inflammation, airways obstruction and emphysema, and is a risk factor for cardiovascular disease (CVD). However, the contribution of these individual COPD components to this increased risk is unknown. Therefore, the aim of this study was to determine the contribution of emphysema in the presence or absence of pulmonary inflammation to the increased risk of CVD, using a mouse model for atherosclerosis. Because smoke is a known risk factor for both COPD and CVD, emphysema was induced by intratracheal instillation of porcine pancreatic elastase (PPE).

**Methods:**

Hyperlipidemic APOE*3-Leiden mice were intratracheally instilled with vehicle, 15 or 30 µg PPE and after 4 weeks, mice received a Western-type diet (WTD). To study the effect of emphysema combined with pulmonary inflammation on atherosclerosis, mice received 30 µg PPE and during WTD feeding, mice were intranasally instilled with vehicle or low-dose lipopolysaccharide (LPS; 1 µg/mouse, twice weekly). After 20 weeks WTD, mice were sacrificed and emphysema, pulmonary inflammation and atherosclerosis were analysed.

**Results:**

Intratracheal PPE administration resulted in a dose-dependent increase in emphysema, whereas atherosclerotic lesion area was not affected by PPE treatment. Additional low-dose intranasal LPS administration induced a low-grade systemic IL-6 response, as compared to vehicle. Combining intratracheal PPE with intranasal LPS instillation significantly increased the number of pulmonary macrophages and neutrophils. Plasma lipids during the study were not different. LPS instillation caused a limited, but significant increase in the atherosclerotic lesion area. This increase was not further enhanced by PPE.

**Conclusion:**

This study shows for the first time that PPE-induced emphysema both in the presence and absence of pulmonary inflammation does not affect atherosclerotic lesion development.

## Introduction

Chronic obstructive pulmonary disease (COPD) is characterized by an excessive inflammatory response towards noxious particles and gases, such as cigarette smoke (CS). In most patients, CS is the main trigger that leads to activation of macrophages and epithelial cells, resulting in the recruitment and activation of other (immune) cells, mucus hypersecretion, alveolar wall destruction (emphysema) and airway remodeling [1]. These structural changes in the airways and lung parenchyma cause the chronic progressive airflow obstruction.

In addition to these pulmonary manifestations, COPD patients often present with comorbidities such as cardiovascular diseases (CVD) and lung cancer. Interestingly, also after correction for common risk factors such as smoking, COPD patients have an increased risk to develop CVD. Atherosclerosis is the main underlying cause of CVD [2], and dyslipidemia and systemic inflammation are considered to be the most important contributors to atherosclerosis development. This increased risk of COPD patients for CVD is thus independent of common risk factors, such as smoking, which can have substantial effects on atherosclerosis development by itself [3], [4]. Current therapy of COPD patients with CVD is based on lipid-lowering treatment which is added to COPD treatment with bronchodilators and anti-inflammatory drugs [5]. However, treatment for COPD patients with CVD is still not optimal, possibly in part because the symptoms are treated as separate modalities and do not take the possible interactions between COPD and CVD into account [5].

Several studies have addressed the link between COPD and CVD, and indicated that persistent low-grade systemic inflammation may be the connection between these two diseases [2], [6]. Indeed, COPD patients show persistent low-grade systemic inflammation as shown by elevated levels of circulating leukocytes, C-reactive protein (CRP), interleukin-6 (IL-6), interleukin-8 (IL-8), tumor necrosis factor-α (TNF-α) and fibrinogen [7], [8]. However, it is difficult to study the contribution of the individual components, as both COPD and CVD are multifactorial and share common risk factors. Furthermore, although this enhanced systemic inflammation appears to be a plausible link between CVD and COPD, little is known about the origin of these circulating inflammatory mediators. Anti-inflammatory therapies in COPD, e.g. inhaled corticosteroids, have not been consistently shown to modify serum levels of CRP or IL-6, nor the long-term cardiovascular complications [9], [10]. This may suggest that local delivery of anti-inflammatory drugs to the lungs does not reduce systemic inflammation. Furthermore, levels of inflammatory mediators (i.e. TNF-α, IL-6 and IL-8) in induced sputum do not correlate with the values of these mediators in blood of COPD patients [11], [12]. Therefore, it is likely that other mechanisms than inflammation are also involved in the interaction between COPD and CVD.

Pulmonary emphysema is a major component of COPD that is present to a variable extent in COPD patients, and several studies have shown that emphysema is associated with all-cause mortality, which also includes respiratory and cardiovascular mortality [13], [14]. Experimental emphysema and atherosclerosis models can help to unravel the interactions between COPD and CVD in patients. CS exposure can be used to induce emphysema in mice, and also has been shown to increase atherosclerosis in *apoe*-/- mice [3], [4]. However, since CS itself also directly affects atherosclerosis, it is a less suitable model to dissect the interaction between COPD and CVD. Intratracheal administration of porcine pancreatic elastase (PPE) in animals also reproduces key phenomena of emphysema. However, in contrast to other challenges that cause emphysema, such as chronic smoke exposure and high-dose intrapulmonary lipopolysaccharide (LPS) instillation [15], PPE induced emphysema is not associated with chronic inflammation, but is characterized by only a transient acute inflammatory response in the lung [16]. This feature of the PPE model allows a separate analysis of the contribution of emphysema and chronic inflammation to atherosclerosis development in experimental models, in absence of the direct effects of cigarette smoking. This is important since it is unknown whether alveolar destruction in the absence of pulmonary inflammation can enhance or contributes to atherosclerosis development. Furthermore, addition of low-dose intranasal LPS administration, which does not further affect emphysema severity, mimics pulmonary inflammation and allows studying the role of PPE-induced emphysema combined with low-grade LPS-induced pulmonary inflammation in atherosclerosis development.

The aim of this study was therefore to assess whether emphysema *per se*, or with concomitant pulmonary inflammation, enhances atherosclerosis development in hyperlipidemic, atherosclerosis-prone APOE*3-Leiden (*E3L*) mice, which represent a well-established model for human-like lipoprotein metabolism, inflammation, and atherosclerosis development [17].

## Materials and Methods

### Animals

Mice were housed under standard conditions with a 12-hour light/dark cycle and had free access to food and water. Female APOE*3-Leiden (*E3L*) mice of 10–12 weeks of age were fed a synthetic diet containing 15% (w/w) cacao butter (diet T; Hope Farms, Woerden, The Netherlands).

Two atherosclerosis studies were performed to examine whether emphysema *per se*, or with concomitant pulmonary inflammation, enhances atherosclerosis development. In the first study, aimed to examine the effect of emphysema alone on atherosclerosis development, mice were fed a run-in diet T for 4 weeks. Subsequently, mice were randomized into 3 groups according to their plasma cholesterol levels, age and body weight, and pulmonary emphysema was induced by intratracheal administration of porcine pancreatic elastase (PPE) (E7885, Sigma-Aldrich, Schnelldorff, Germany) (15 µg or 30 µg in 40 µL PBS) [18], [19]. Control mice received 40 µl sterile PBS (vehicle). In the second study, in order to examine the effect of emphysema combined with chronic low-grade pulmonary inflammation on atherosclerosis development, 30 µg PPE in 40 µL PBS or 40 µL PBS as control was administered intratracheally to induce pulmonary emphysema, after run-in diet feeding and randomization.

In both studies, mice were allowed to recover from the PPE-instillation for 4 weeks, and subsequently were fed a Western-type diet (WTD) [20] to induce atherosclerosis development. During these 20 weeks, mice in the second study received 1 µg LPS (serotype 055:B5 *Escherichia coli* LPS) in 50 µl PBS intranasally twice weekly. Control mice received 50 µl sterile PBS (vehicle).

At baseline (before intratracheal PPE instillation) and every 4 weeks thereafter, blood was drawn in EDTA-coated tubes (Sarstedt, Numbrecht, Germany) in mice of both studies by tail bleeding after 4 hours fasting, and plasma was isolated by centrifugation. After 20 weeks of WTD feeding, mice were anesthetized by intraperitoneal injection of 6.25 mg/kg acepromazine (Alfasan, Woerden, The Netherlands), 6.25 mg/kg midazolam (Roche, Mijdrecht, The Netherlands), and 0.31 mg/kg fentanyl (Janssen-Cilag, Tilburg, The Netherlands). Subsequently, mice were sacrificed by cervical dislocation and the pulmonary and systemic circulation was rinsed with ice-cold PBS. Lungs were fixed *in situ* by gentle infusion of fixative (phosphate-buffered 4% formaldehyde) by a continuous-release pump under constant pressure (12 mL/hour; 8 min) through a tracheal cannula. After excision, the lungs and heart were immersed in fresh fixative for a period of 24 hours at 4 degrees. Other organs were isolated and stored at −80°C for further analysis.

Both animal experiments were performed once and were approved by the Institutional Ethical Committee on Animal Care and Experimentation of the Leiden University Medical Center (Leiden, The Netherlands).

### Plasma lipids, glucose levels and systemic inflammation analysis

Plasma total cholesterol (TC), triglyceride (TG) and phospholipid (PL) levels were determined using enzymatic kits from Roche Molecular Biochemicals (Woerden, The Netherlands). Plasma glucose levels were measured using enzymatic kits from Instruchemie (Delfzijl, The Netherlands); all analyses were performed according to the manufacturer's protocols.

Plasma levels of serum amyloid A (SAA), soluble E-selectin (sE-selectin), interleukin-6 (IL-6) and tumour-necrosis factor-α (TNF-α) were determined using the murine SAA assay kit (Tridelta, County Kildare, Ireland), murine E-selectin ELISA kit (R&D, Minneapolis, MN), murine IL-6 kit (BD Biosciences Pharmingen) and murine TNF ELISA (Bender MedSystems) respectively, according to manufacturer's instructions.

### Pulmonary function measurements

Respiratory depth and rate were assessed 13 weeks (first study) and 8 and 18 weeks (second study) after instillation of vehicle or PPE with whole-body plethysmography (RM-80, Columbus Instruments, Columbus, OH, USA) in freely moving mice, as described previously [21]. The signal was digitized using a Digidata 1440 A interface (Axon Instruments/Molecular Devices, Union City, CA, USA) and analyzed with the event detection feature of Clampfit 9.2 (Axon Instruments/Molecular Devices). The average peak-to-peak amplitude of the signal, representing a measure for tidal volume, was determined from the signal recorded during a two minute period.

### Histological analyses of the lungs

Lungs were processed for paraffin embedding and cut in 5 µm coronal sections. Tissue samples were stained with hematoxylin-eosin (HE). To assess air space enlargement, the mean linear intercept (MLI) and air/tissue (AT) ratio was quantified by one observer in a blinded fashion by superimposing a line grid with 21 lines and 42 points on the images of lung sections at a magnification of 200× as described previously [22]. To calculate the MLI, the number of intersections between the lines of the grid and the alveolar walls was quantified for each mouse in 10 non-overlapping fields. To determine the air/tissue ratio, the number of points in alveolar space was counted.

For immunohistochemical staining, slides were pretreated with 1% H_2_O_2_ in methanol to block endogenous peroxidase activity. Sections were incubated overnight with rat anti-mouse MAC-3 antibody (1∶50, BD Pharmingen, Breda, The Netherlands) (study 1), rat-anti-mouse F4/80 antibody (1∶60) (study 2), or anti-myeloperoxidase (MPO) (1∶1500, Thermo Fisher scientific, Runcorn, United Kingdom) to detect macrophages [23] and neutrophils [22] respectively in the lung, and washed with PBS/0.05% Tween-20. Biotinylated anti-rat antibody (1∶300, GE Healthcare Amersham, Buckinghamshire, United Kingdom) or anti-rabbit EnVision-HRP was added, respectively, and incubated for 30 minutes. Subsequently, slides were washed with PBS/0.05% Tween-20 and peroxidase-streptavidine was applied to the slides for the MAC-3 or F4/80 staining, and incubated for 30 minutes. After washing with PBS/0.05% Tween-20, Nova Red substrate (Vector Laboratories Inc., Burlingame, CA) was added and incubated for 5 minutes. Slides were rinsed and counterstained with hematoxylin.

Results of MAC-3, F4/80 and MPO positive cells are represented as the average count from 10 non-overlapping fields per mouse (40× magnification) corrected for tissue density by calculating the ratio between the number of cells and the average area of tissue per field. To determine the average area of tissue, the number of points superimposed on alveolar tissue was counted (40× magnification), using the same line grid for assessment of air space enlargement, as described above.

### Histological analyses of the heart

Hearts were isolated and fixed in phosphate-buffered 4% formaldehyde and processed for paraffin embedding. A 5 µm transversal section of the heart halfway in the long axis was stained with HE. Thickness of the right and left ventricular free walls was assessed at a 40× magnification by averaging 6 measurements per structure with the NIH Image J program.

For quantification and classification of atherosclerosis, the hearts were cross-sectioned (5 µm) throughout the entire aortic root area. Per mouse, 4 sections with 40-µm intervals were used for quantification of atherosclerotic lesion area and characterization of lesion severity by after staining with hematoxylin-phloxine-saffron (HPS). Atherosclerotic lesions were categorized for severity, according to the guidelines of the American Heart Association [17], adapted for mice. The segments were categorized into: 1) no lesions 2) mild (type I-III) and 3) severe (type IV-V) lesions.

Immunohistochemistry for determination of adhering monocytes and macrophage-, smooth muscle cell content in the lesions was performed as described previously [24]. In brief, the sections were blocked in 0.3% H2O2 in methanol. After overnight incubation with AIA31240 rabbit antiserum (1∶1000, Accurate Chemical and Scientific, Westbury, NY) for quantification of the number of monocytes adhering to the endothelium and the macrophage area, or mouse monoclonal antibody M0851 (1∶800, Dako, Carpinteria, CA) for quantification of smooth muscle actin, biotinylated donkey anti-rabbit conjugate (Amersham Pharmacia Biotech, Roosendaal, The Netherlands) or biotinylated horse anti-mouse conjugate, respectively, was added (Vector Laboratories Inc., Burlingame, CA). Immunostaining was amplified using Vector Laboratories Elite ABC kit (Vector Laboratories Inc., Burlingame, CA) and the immunoperoxidase complex was visualized with Nova Red (Vector Laboratories Inc., Burlingame, CA). Counterstaining was performed with hematoxylin. Sirius red (Chroma, Stuttgart, Germany) was used to stain for collagen in the lesions.

Total lesion area, macrophage -, smooth muscle cell – and collagen content were quantified using Cell∧D image analysis software (Olympus Soft Imaging Solutions, Münster, Germany). Fibrillar collagen (collagen type I + type III) were analyzed on Sirius red-stained sections using polarized light microscopy.

### Statistical analysis

Statistical significance of differences was assessed with one-way ANOVA analysis, followed by post-hoc analysis using Fisher's LSD multiple comparison test. To evaluate the interaction between PPE and LPS in the second study, two-way ANOVA was used. Bodyweight and lipid parameters were analyzed using two-way ANOVA test for repeated measurements. Differences between groups were further explored using one-way ANOVA and post-hoc analyses as described above.

Differences at *p*<0.05 were regarded as statistically significant. Data are presented as means ± SEM.

## Results

### Intratracheal administration of PPE induces emphysema dose-dependently

**Figure 1:**
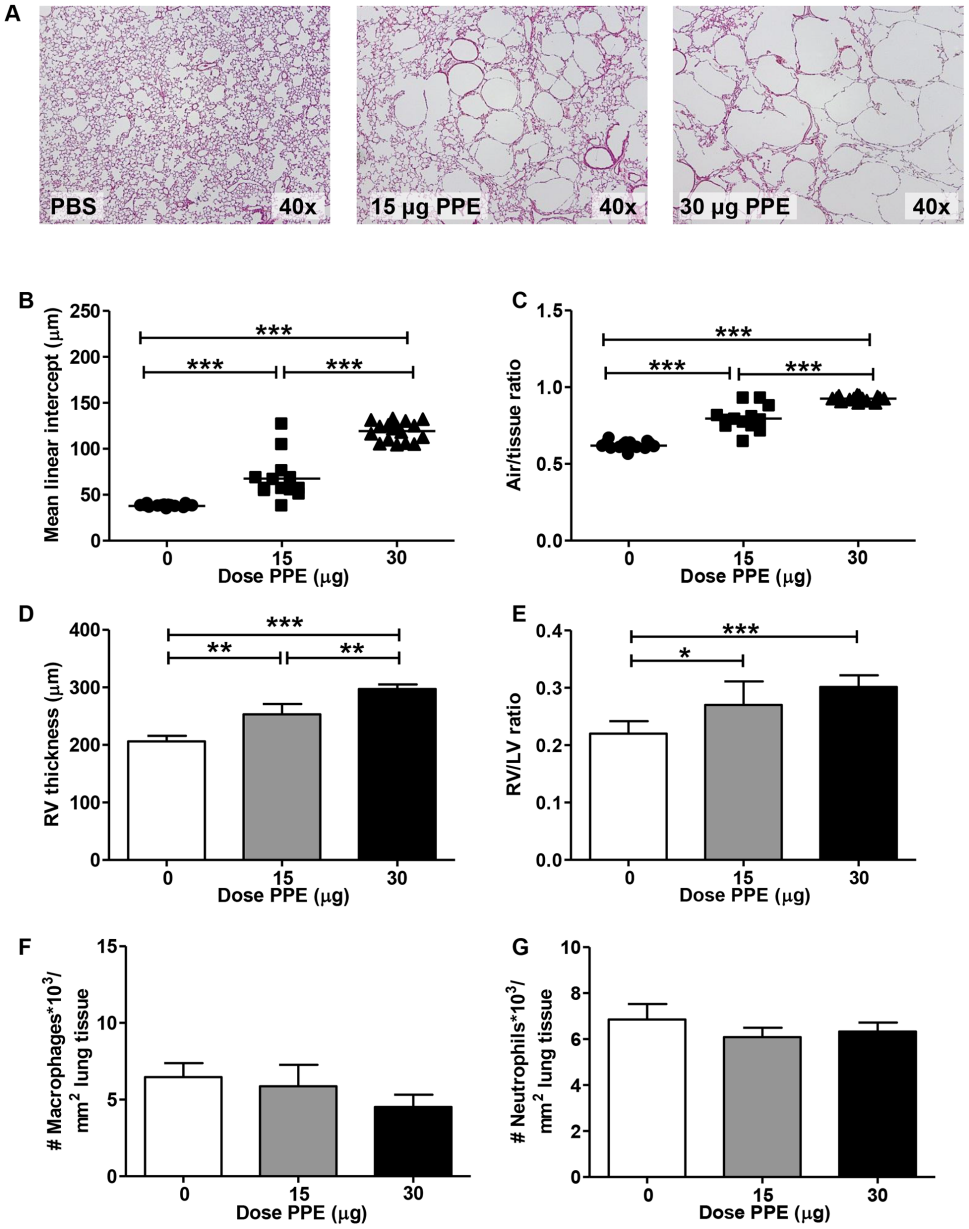
Intratracheal PPE instillation dose-dependently increases alveolar destruction, without affecting pulmonary inflammation. E3L mice were intratracheally instilled with vehicle, 15 or 30 µg PPE and sacrificed after 24 weeks. Mean linear intercept (B) and air/tissue ratio (C) were determined on sections of the lungs (A). Furthermore, right ventricular (RV) (D) and left ventricular (LV) wall thickness was determined and the ratio was calculated (E). The number of MAC3-positive macrophages (F) and MPO-positive neutrophils (G) in the lung was determined immunohistochemically. Values in B+C are represented as the mean; values in D-G are presented as means ± SEM; n = 12–15; *p<0.05, **p<0.01, ***p<0.001.

To determine the contribution of emphysema alone on atherosclerosis development, *E3L* mice were intratracheally instilled with vehicle or 15 or 30 µg PPE after which atherosclerosis was induced by 20 weeks of WTD feeding. Compared to vehicle, intratracheal PPE instillation caused a significant dose-dependent increase in emphysema (representative images are shown in Fig. 1A) as determined by morphometrical assessment of the MLI (Fig. 1B) and AT ratio (Fig. 1C), indicating destruction of alveolar walls and enlargement of alveolar space. In addition to development of emphysema, a dose-dependent increase in right ventricular hypertrophy was observed with a single dose of PPE (Fig. 1D and E), indicating pulmonary hypertension which is often observed in COPD patients.

After 20 weeks WTD diet, there was no difference between PPE-treated mice and controls in pulmonary inflammatory cell influx as measured by the number of macrophages and neutrophils in lung tissue (Fig. 1F and G).

### Intratracheal administration of PPE does not affect plasma inflammatory parameters, plasma lipids and atherosclerotic lesion area

Dyslipidemia and systemic inflammation are important contributors to atherosclerosis development. Therefore, we measured the acute-phase proteins SAA and sE-selectin as markers for systemic inflammation and endothelial activation. These parameters were not affected by PPE-induced inflammation during the study (not shown). Furthermore, plasma TC (Fig. 1A), TG and PL (not shown) levels were similar between all groups.

**Figure 2:**
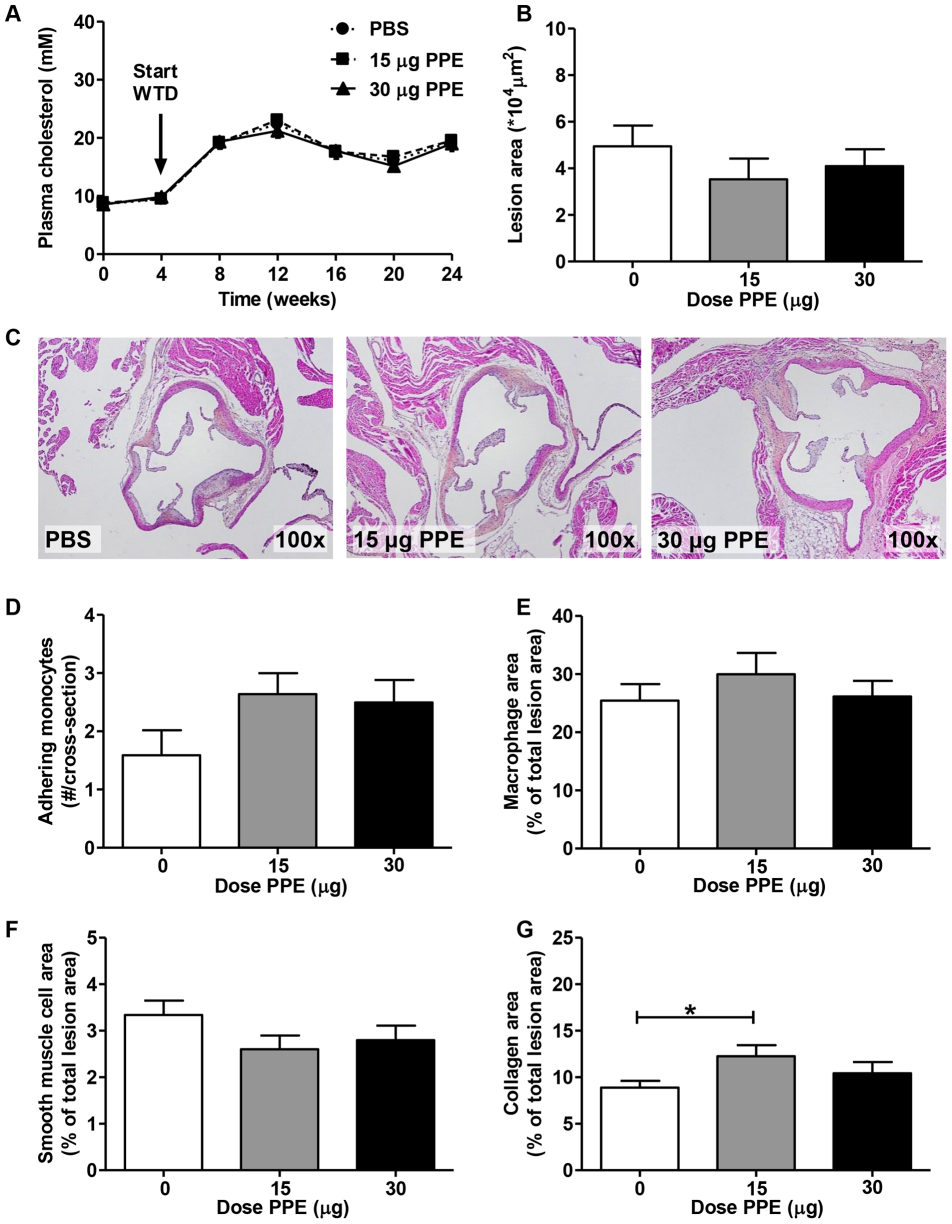
PPE-induced emphysema does not affect atherosclerosis lesion area. During the study, blood was drawn to assess plasma cholesterol levels (A). After 20 weeks of WTD feeding mice were sacrificed, hearts were isolated and atherosclerotic lesion area (B) was determined in the aortic root area (representative pictures are shown in C). Also, the number of monocytes adhering to the vessel wall (D) and macrophage- (E), smooth muscle cell- (F) and collagen (G) content of the atherosclerotic lesions were measured. Values are presented as means ± SEM; n = 12–15; *p<0.05.

To determine the effect of PPE-induced emphysema on atherosclerosis development, we analyzed atherosclerosis development in the aortic root (representative images are shown in Fig. 2C) by assessing total atherosclerotic lesion area and composition. No differences were observed in atherosclerotic lesion size (Fig. 2B), the number of monocytes adhering to the vessel wall (Fig 2D), macrophage lesion content (Fig. 2E) or smooth muscle cell lesion content (Fig. 2F) between PPE- and control-treated mice. The group treated with 15 µg PPE had a significantly higher collagen content in the lesions (Fig. 2G) compared to vehicle, which could not be attributed to a difference in collagen type I+III content of the Sirius red-positive area of the lesions.

### PPE-induced emphysema combined with low-dose intranasal LPS administration induces chronic pulmonary inflammation, without affecting emphysema development

Since PPE-induced emphysema alone did not affect atherosclerosis development, we assessed the effect of PPE-induced emphysema combined with chronic low-grade LPS-induced pulmonary inflammation on atherosclerosis development in a second study. Therefore, emphysema was induced by Intratracheal instillation of 30 µg PPE in *E3L* mice and subsequently, the mice were instilled with a low dose of LPS (1 µg) twice weekly during 20 weeks WTD feeding to induce pulmonary inflammation.

**Figure 3:**
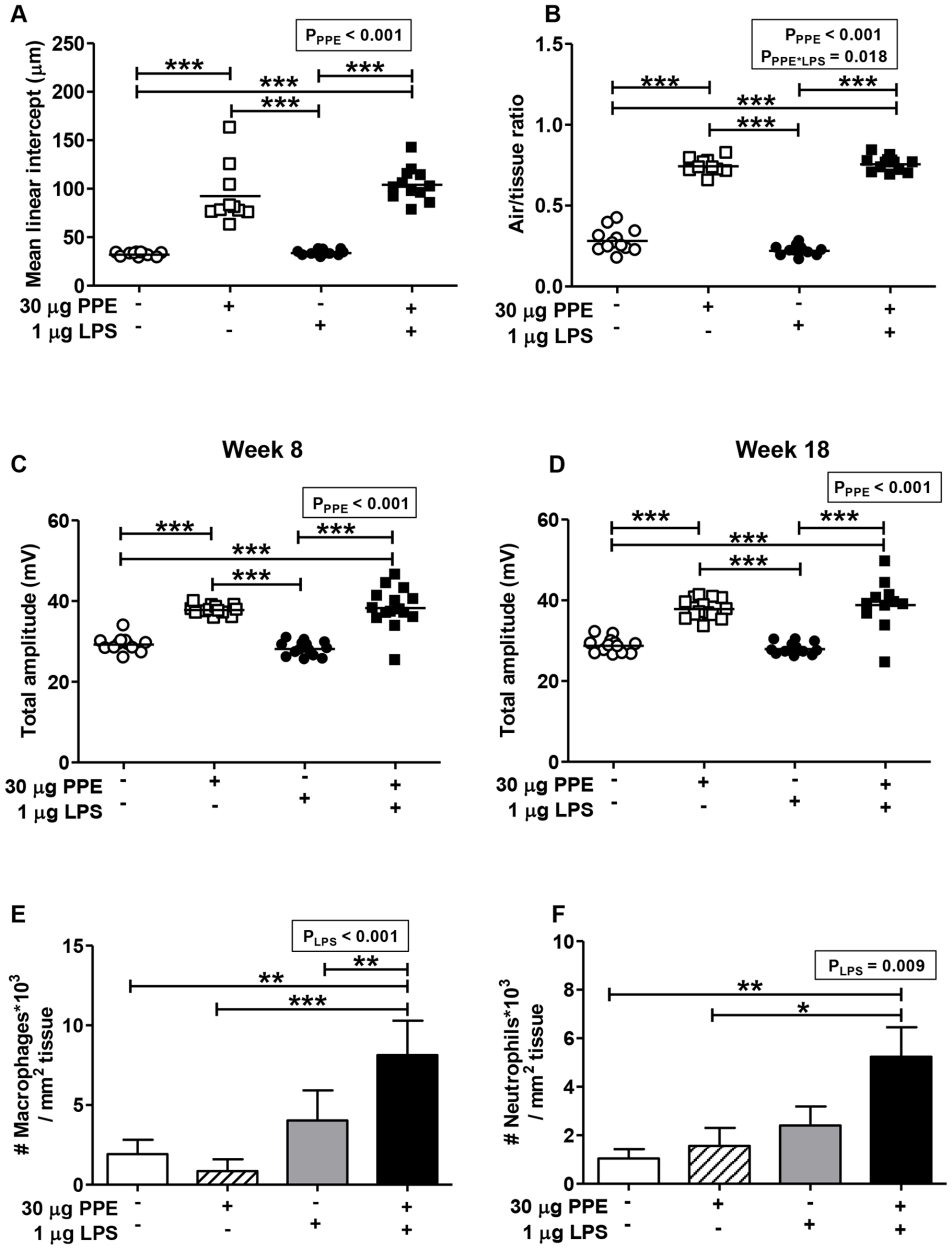
PPE-induced emphysema combined with low-dose LPS administration induces chronic low-grade pulmonary inflammation, without affecting emphysema. After intratracheal instillation of vehicle or PPE (30 µg/mouse) in *E3L* mice, low-dose intranasal LPS (1 µg/mouse) or vehicle was administered during 20 weeks WTD feeding. Total respiratory amplitude was analyzed at 8 and 18 weeks (C and D) after PPE. After sacrifice, mean linear intercept (A) and air/tissue ratio (B) were determined and inflammatory cell influx was determined by immunohistochemical staining of macrophages with F4/80 (E) and neutrophils with MPO (F). Significant effects detected by two-way ANOVA analysis are shown as p-value in textboxes above the figure. Values are presented as means ± SEM; n = 10–15; *p<0.05, **p<0.01, ***p<0.001.

Similar as in the first study (Fig. 1A–C), intratracheal PPE instillation induced emphysema as indicated by a significant increase in MLI (Fig. 3A), AT ratio (Fig. 3B) and respiratory amplitude (Fig. 3C and D). As anticipated, repeated low-dose intranasal LPS instillation did not affect any of these parameters.

To measure the extent of pulmonary inflammation, the number of macrophages and neutrophils were quantified by immunohistochemistry. Two-way ANOVA analyses showed that exposure to LPS significantly increased both pulmonary macrophages (pLPS<0.001, pPPE = 0.315, pPPE*LPS = 0.068) and neutrophils (pLPS = 0.009, pPPE = 0.075, pPPE*LPS = 0.217), indicating that LPS instillation irrespective of PPE treatment increased pulmonary inflammation. Further analyses showed that PPE-induced emphysema did not increase the number of pulmonary macrophages (Fig. 3E) and neutrophils (Fig. 3F) in the absence of intranasal LPS instillation, confirming the results from the first study. However, PPE-induced emphysema in combination with LPS instillation significantly increased the number of pulmonary macrophages as compared to LPS instillation alone, and tended to increase the number of neutrophils, although this did not reach statistical significance. These findings indicate that low-dose intranasal LPS instillation modestly increased pulmonary inflammation, which was further enhanced by PPE-induced emphysema.

### PPE-induced emphysema combined with chronic LPS-induced pulmonary inflammation induces limited systemic inflammation and does not affect plasma lipid levels

**Figure 4:**
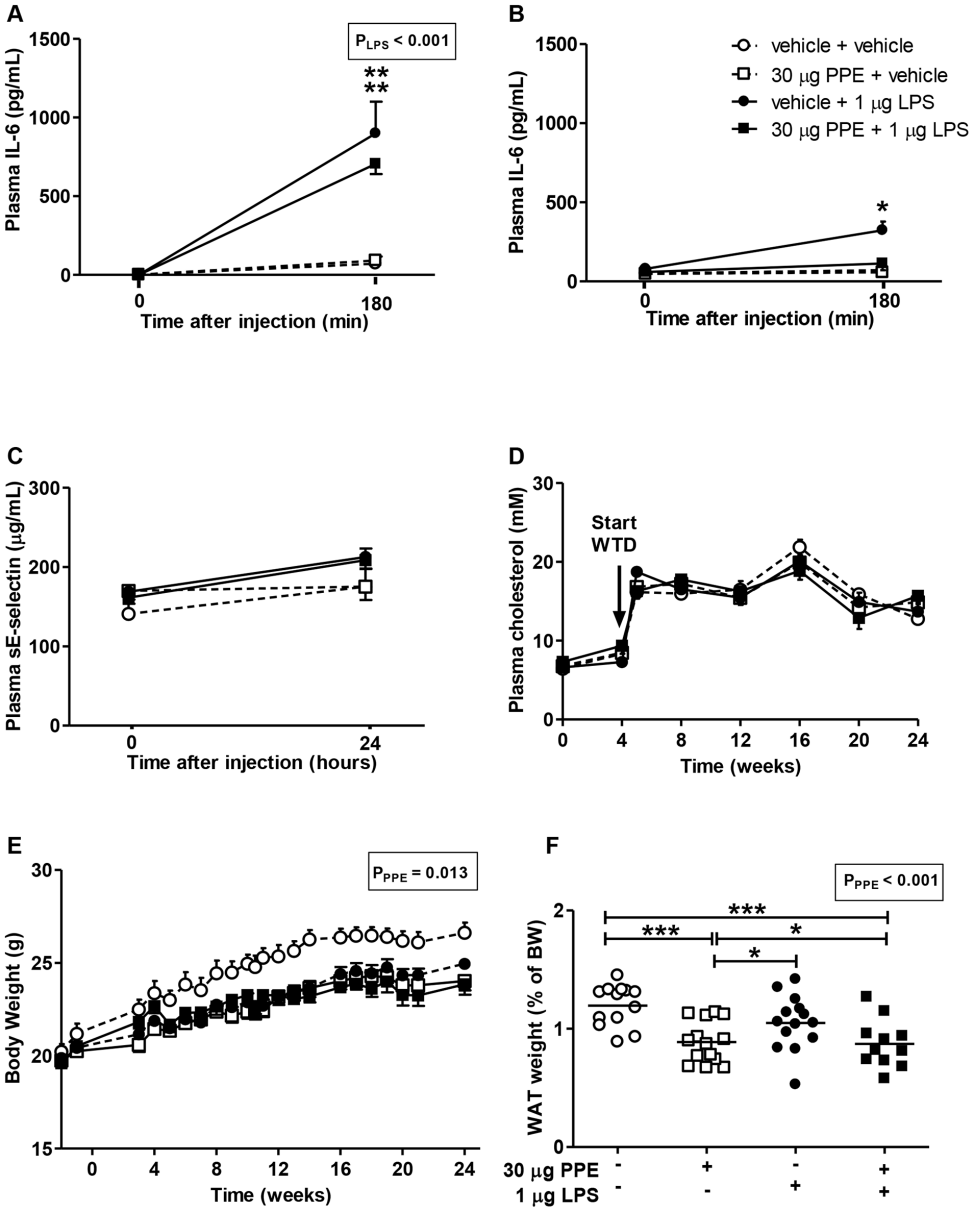
Low-dose intranasal LPS administration induces low-grade systemic inflammation and does not affect plasma lipid levels. Plasma IL-6 levels were determined at 0 and 180 min after the first LPS administration (A) and again after the LPS administration in week 18 (B). Plasma sE-selectin levels were measured 24 hours after the first LPS instillation (C). Plasma total cholesterol levels (D) and body weight (E) were monitored during the study. After 20 weeks WTD, mice were sacrificed and white adipose tissue (WAT) weight was determined (F). Significant effects detected by two-way ANOVA analysis are shown as p-value in textboxes above the figure. Values in A–E are presented as means ± SEM and values in F are presented as means; n = 10–15; *p<0.05, **p<0.01.

The first intranasal instillation of LPS induced a low, but evident systemic inflammatory response, as measured by increased plasma IL-6 levels (Fig. 4A). After repeated instillation, only mice treated with LPS alone showed a low systemic IL-6 response (Fig. 4B), which was not observed in mice with PPE-induced emphysema combined with intranasal LPS administration. Plasma sE-selectin levels were not different between groups 24 h after the first LPS administration (Fig. 4C). Plasma TNF-α levels were not detectable after intranasal LPS instillation (not shown).

Plasma TC (Fig. 4D), as well as TG, PL and glucose (not shown) levels were similar between groups throughout the study. During the study, mice treated with PPE showed a lower body weight as compared to the control group (Fig 4E). Concomitant with this, white adipose tissue weight was lower in PPE treated mice as compared to control (Fig. 4F).

### PPE-induced emphysema combined with chronic low-grade LPS-induced pulmonary inflammation does not affect atherosclerotic lesion area and severity

**Figure 5:**
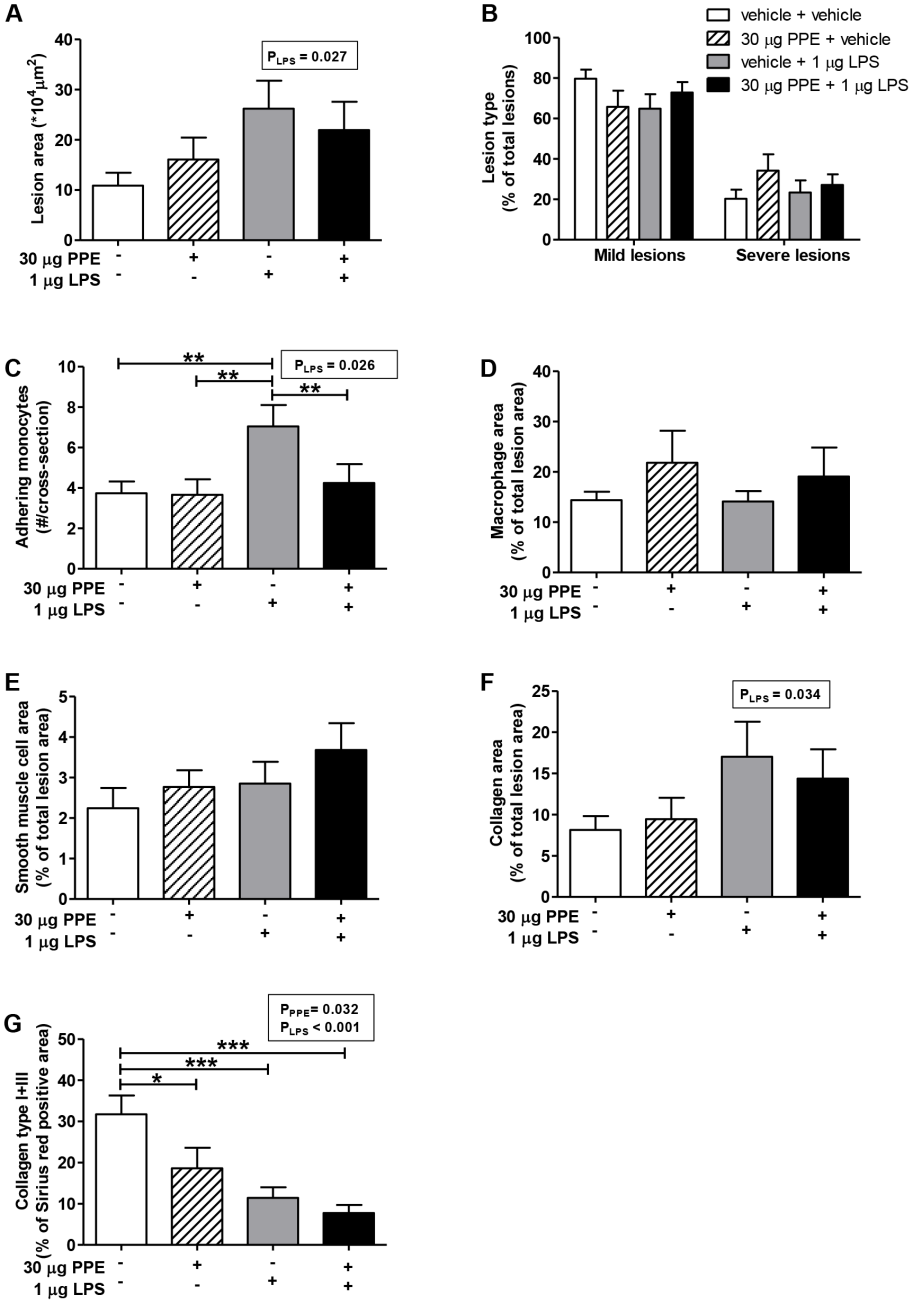
PPE-induced emphysema combined with chronic low-grade LPS-induced pulmonary inflammation does not affect atherosclerosis development. After 20 weeks of WTD feeding, mice were sacrificed and the total atherosclerotic lesion area (A) and lesion severity (B) were assessed. Furthermore, the number of monocytes adhering to the vessel wall, (C) macrophage (D), smooth muscle cell (E), collagen content (F) of the atherosclerotic lesions and collagen type I+III of Sirius red-positive area were measured. Significant effects detected by two-way ANOVA analysis are shown as p-value in textboxes above the figure. Values are presented as means ± SEM; n = 10–15; *p<0.05, **p<0.01, ***p<0.001.

To study the effect of PPE-induced emphysema combined with chronic LPS-induced pulmonary inflammation on atherosclerosis development, we assessed atherosclerotic lesion area (Fig. 5A), severity (Fig. 5B) and composition (Fig 5C–G) in the aortic root. Two-way analyses showed that intranasal LPS instillation increased atherosclerotic lesion area (pLPS = 0.027, pPPE = 0.919, pPPE*LPS = 0.313), which was not further aggravated by PPE-induced emphysema. Furthermore, LPS administration increased adherence of monocytes to the vessel wall in the aortic root area (Fig. 5C). Lesion macrophage (Fig. 5D) and smooth muscle cell (Fig 5E) content were not different between groups. Intranasal LPS instillation significantly increased total collagen content of the atherosclerotic lesion (Fig 5F; pLPS = 0,034, pPPE = 0.831, pPPE*LPS = 0.531). Interestingly, the contribution of collagen type I+III to the total Sirius red-positive area decreased significantly after treatment with PPE, LPS or a combination of PPE and LPS compared to vehicle (Fig. 5G; pPPE = 0.032, pLPS<0.001 and pPPE*LPS = 0.220). The increased collagen content is thus most likely explained by an increase in collagen types other than type I+III.

## Discussion

COPD patients have an increased risk to develop CVD even after correction for common risk factors such as smoking, but the exact pathophysiological link between COPD and CVD is unknown. Using a PPE-induced experimental emphysema model, which is not accompanied by chronic local or systemic inflammation, we show for the first time that emphysema alone does not affect atherosclerosis development. Furthermore, we demonstrate that PPE-induced emphysema combined with repeated low-dose intranasal LPS administration in *E3L* mice enhanced pulmonary and systemic inflammation, and although LPS exposure caused a limited, but significant increase in the atherosclerotic lesion area, this was not further enhanced by intratracheal PPE-induced emphysema.

Several studies have explored the effect of exposure to CS (the main risk factor for COPD) on atherosclerosis development. CS exposure may have direct and indirect effects on atherosclerosis development by affecting plasma lipids and systemic inflammatory parameters. Han *et al.* [25], for example, showed that 15 weeks exposure to sidestream CS in both *apoe* ^-/-^ and *ldlr* ^-/-^ mice resulted in increased atherosclerotic lesion area, which in *ldlr* ^/-^ mice was due to increased plasma cholesterol levels. Exposure to sidestream CS also resulted in increased atherosclerotic lesion area and in increased plasma cholesterol levels in *apoe* ^-/-^ mice [3]. However, in these studies, the role of CS-induced (pulmonary and systemic) inflammation was not examined. Arunachalam *et al.* [26] showed that exposure to CS in *apoe* ^-/-^ mice induced pulmonary inflammation, as assessed by increased inflammatory cell influx and inflammatory cytokines. However, in this study the effect on atherosclerosis was not examined. Collectively these studies in hyperlipidemic mice show that long-term CS exposure increases atherosclerotic lesion area, most likely due to effects on plasma lipid levels [3], [25], but do not provide an explanation for the link between COPD and CVD. Therefore our study contributes to the existing information by showing that emphysema *per se* does not affect atherosclerosis development.

In order to delineate the putative contribution of several features of COPD to atherosclerosis development, we first induced emphysema by intratracheal PPE instillation in *E3L* mice and, subsequently, initiated atherosclerosis formation by WTD feeding. In line with other studies, we observed that instillation of PPE dose-dependently induced emphysema [16], without affecting the number of inflammatory cells (*i.e.* macrophages and neutrophils) in the lungs. In addition to the absence of pulmonary inflammation, emphysema also did not cause systemic inflammation (*i.e.* plasma SAA and sE-selectin) during the study. Therefore, we next assessed whether chronic low-grade LPS-induced pulmonary inflammation and the accompanying low-grade systemic inflammation enhanced atherosclerosis development. We deliberately used a low dose of LPS (1 µg/mouse) to prevent further LPS-induced alveolar destruction, since several studies showed that high-dose intranasal or intratracheal LPS administration (ranging from 5–25 µg/mouse) can cause alveolar destruction [15]. Furthermore, Westerterp *et al.* showed that weekly intraperitoneal injection of high-dose LPS can cause a chronic inflammatory state which aggravates atherosclerosis development, *e.g.* by direct effects on the arterial wall [24]. This suggests that intraperitoneal injection of LPS might have differently affected atherosclerosis development in our study, as compared to the currently used intranasal administration. However, in our opinion, the direct interaction between PPE-induced emphysema and pulmonary inflammation as a model for COPD cannot be properly studied using intraperitoneal LPS injections.

The first intranasal LPS instillation induced a low but evident systemic IL-6 response, both in mice exposed to LPS and with PPE combined with LPS. Repeated LPS instillation, continued to induce a very low systemic IL-6 response; however, this was not observed in mice with PPE-induced emphysema. This reduced IL-6 response after repeated instillation is most likely caused by the occurrence of LPS-induced tolerance often observed in such studies [27].

It has been hypothesized that spillover of lung-derived inflammatory mediators in the systemic circulation explains the increased risk of CVD in COPD patients [2]. This is supported by experimental studies which have separately shown that increased plasma IL-6 may modulate the plaque composition through spill-over from the lungs. Tamagawa *et al.* [28] showed that IL-6 was translocated from the lungs to the systemic circulation, as shown by a difference in arteriovenous IL-6 levels due to increased lung inflammation and concomitant lung permeability. Studies by others have shown that systemic IL-6 contributes to atherosclerosis development [29]. This is in line with our findings that increased plasma IL-6 is accompanied by an increase in lesion area in the mice that received LPS.

Other systemic inflammatory markers such as sE-selectin and SAA were unaffected and a TNF-α response was not detectable. Using TNF-α knockout mice on a *E3L*-background, Boesten *et al.* [30] showed that while the absence of TNF-α did not affect plasma lipid and inflammatory parameters, it did reduce lesion severity, suggesting that TNF-α is a pro-atherogenic cytokine in atherosclerosis development. TNF-α is thought to decrease plaque stability through increased necrosis and matrix protease activation [30]. Therefore, the absence of increased circulating TNF-α levels in our study may help to explain why atherosclerosis development was only mildly increased. However, it cannot be excluded that other inflammatory mediators were increased in our studies.

In addition to systemic inflammation, increased plasma cholesterol levels are important contributors to atherosclerosis development in the CS models discussed. Hypercholesterolemia, with increased circulating levels of low-density lipoprotein (LDL)- and/or very-low-density lipoprotein (VLDL)-cholesterol, is a well-established risk factor for atherosclerosis development and progression [17]. CS mostly affects plasma lipids by oxidation, thereby enabling increased foam cell formation in the vessel wall. In the present study, PPE-induced emphysema did not affect plasma lipid parameters. The lack of systemic inflammation and unaffected plasma lipids may help explain why emphysema did not affect atherosclerosis development in *E3L* mice.

Another potential mechanism linking COPD to enhanced CVD is chronic hypoxia [31]. PPE-treated mice also showed signs of chronic hypoxia, as indicated by a significantly higher number of circulating erythrocytes, hemoglobin and hematocrit levels (not shown). Although hypoxia can promote atherogenesis [31], PPE instillation did not affect lesion severity or area. It is most likely that the magnitude of hypoxia in the PPE-treated mice was compensated by 1) an increased respiration amplitude, which probably is a compensatory mechanism for the reduction in diffusion area; and 2) an increase in the number of circulating erythrocytes. Moreover, the development of right ventricular hypertrophy as observed in the present study, and reported in COPD patients by others [32], may also have acted as a compensatory mechanism. Therefore, in the PPE-treated mice it is likely that the level of hypoxia was minimized by these compensatory mechanisms.

One of the risk factors for the development of atherosclerosis is obesity [33], which is also associated with chronic low-grade inflammation. Secretion of pro-inflammatory adipokines may contribute to the low-grade systemic inflammatory phenotype often observed in COPD patients. COPD patients have a high prevalence of abdominal obesity as compared to age and sex-matched controls. Adipose tissue-derived pro-inflammatory adipokines combined with inflammatory mediators derived from the lung may contribute to the systemic inflammatory phenotype [33]. In the present study, mice which were instilled with PPE, showed a lower body weight and white adipose tissue mass as compared to the control group. This is in line with the observation that CT-assessed emphysema (as opposed to bronchitis assessed by airway wall thickness) is associated with a decreased fat-free mass [34], [35], although also patients with this COPD phenotype have an increased risk of CVD, most likely caused by several factors such as impaired lung function, increased systemic inflammation and increased oxidative stress.

Finally, intranasal LPS administration increased the total collagen content of the atherosclerotic lesions, which was not paralleled by a difference in SMC or macrophage content. Although we did not determine the activation state of the macrophages and SMCs in the lesions, this finding may indicate that the increased collagen content was not the result of a general increased production of collagen, nor by a general decreased degradation of the collagen by matrix metalloproteases (MMPs). Interestingly, quantification of fibrillar collagen distribution revealed that the increase in collagen content in the lesion could not be attributed to collagen type I and III. Changes in collagen types in the lesions can be due to a difference in the microenvironment (*i.e.* cytokines and chemokines and hemodynamic changes). Furthermore, the production of other extracellular matrix molecules (*i.e.* other collagen types) may have contributed to the increased Sirius red-positive content of the atherosclerotic lesion [36]–[39]. Also, changes in the phenotype of SMCs and production of collagen can occur during chronic pulmonary hypertension and right ventricular hypertrophy [40]–[43]. Mice treated with PPE in both studies showed right ventricular hypertrophy, which may have contributed to a difference in collagen content in the second study, but this was not paralleled by a difference in collagen type I and III in the first study.

In summary, our results indicate that in atherosclerosis-prone mice, intranasal low-dose LPS administration increases pulmonary inflammation and atherosclerotic lesion area, but that emphysema *per se* in the absence or presence of pulmonary inflammation does not aggravate the development of atherosclerosis. Therefore, therapy of CVD in COPD patients should focus on lowering (pulmonary and systemic) inflammation, rather than emphysema.
